# Supplementary material for: Radiologic versus Segmentation Measurements to Quantify Wilms Tumor Volume on MRI in Pediatric Patients
Source: Cancers (Basel). 2023 Apr 1;15(7):2115. doi: 10.3390/cancers15072115 (PMC10092966; doi:10.3390/cancers15072115)
Supplement: Supplementary file 1 [file cancers-15-02115-s001.zip › cancers-2268916-supplementary.pdf]

Supplemental Table S1: Overview of postoperative treatment for localized Wilms tumor in UMBRELLA SIOP-RTSG 2016.

Disease	Tumor volume after pre-operativ chemotherapy	Treatment		
		Stage I	Stage II	Stage III
Low-Risk	All	None	AV (27 weeks)	AV (27 weeks)
Intermediate risk- all subtypes	< 500 ml	AV (4 weeks)	AV (27 weeks)	AV (27 weeks) + flank radiotherapy
Intermediate-risk, stromal or epithelial type	≥ 500 ml	AV (4 weeks)	AV (27 weeks)	AV (27 weeks) + flank radiotherapy
Intermediate-risk, nonstromal, nonepithelial	≥ 500 ml	AV (4 weeks)	AVD (27 weeks)	AV (27 weeks) + flank radiotherapy
High-risk blastemal type	All	AVD (27 weeks)	HR-1 (34 weeks)	HR-1 (34 weeks) + flank radiotherapy
High-risk diffuse anaplasie	All	AVD (27 weeks)	HR-1 (34 weeks) + flank radiotherapy	HR-1 (34 weeks) + flank radiotherapy

A: actinomycin D; D: doxorubicin; HR-1: etoposide, carboplatin, cyclophosphamide and doxorubicin; V: vincristine. Table base on: van den Heuvel-Eibrink et al.[5]
